# Ergot of Rye

**Published:** 1831-04-01

**Authors:** 


### VI. Ergot of Rye.

Drs. Spairani and Pignacea, of Milan, have recently made some observations on the use of the above article, not only in uterine but in other hæmorrhages. Led by analogy, the former of these gentlemen was induced to exhibit the ergot in uterine congestion and hæmorrhage, unconnected with pregnancy or parturition; and as useful in these cases, he determined to extend its exhibition to hæmorrhage in other organs than the uterus—as, for example, in hæmoptysis, epistaxis, and hæmaturia. Having reason to know that the ergot is of immense utility in uterine hæmorrhage, and that it is not so generally administered as it ought to be upon such occasions, we shall devote a page or two to the paper now under view.

*Case* 1. Metrorrhagia. This was a young lady who had been very irregular for some time, and occasionally went two or three months without menstruating, when a profuse discharge would come on. In the present instance it amounted to menorrhagia, and resisted all the usual means. The narrator, Spairani, administered three drachms of the ergot in two days, the dose being about seven or eight grains at a time. The hæmorrhage soon ceased.

*Case* 2. This was a female, who was safely delivered of her fourth child, and the lochia ceased at the expiration of eight days. Soon afterwards the discharge re-appeared, and increased gradually till large clots of blood were daily evacuated. The patient became sleepless, debilitated, and affected with violent pains in her limbs, loins, and hypogastric region. A drachm of the ergot was divided into eight doses, and administered in the 24 hours. The discharge ceased at the end of the first day, and did not re-appear.

*Case* 3. Madam N. was affected with profuse menorrhagia, attended with pains in the loins and hypogastrium. The discharge was pure blood, and had continued for 15 days. The ergot was administered in the manner above-mentioned, and by the end of the second day she was quite well.

*Case* 4. Madame T. had been confined of a child two months, when, without any evident cause, a profuse menorrhagia came on, and continued for six days, rather augmenting than diminishing. Nevertheless the health was excellent, and no pains accompanied the discharge. Eight grains of the ergot were ordered every two hours. On the next day, the discharge was considerably diminished, and on the third day it entirely disappeared.

*Case* 5. Judith Massana, aged 36 years, had borne five children safely, and one laboriously. It was a few months after this last accouchement, that the patient was seized with menorrhagia, which augmented in quantity, and was mingled with large clots of blood. Fifteen grains of the ergot were administered twice a day for two days, when the discharge entirely ceased. The remedy, however, was continued a few days longer.

*Case* 6. N. N. aged 27 years, aborted frequently, and had had frequent attacks of menorrhagia. The uterus was enlarged, and there was a suspicious excrescence on the left side of the cervix uteri. Our author was called to her on the twentieth day of a profuse uterine discharge, and by which she was reduced to a deplorable state of pallor and debility; she was also suffering greatly from pains in the loins and neighbouring parts. The ergot was administered in infusion, and the discharge was checked within twenty-four hours. On the third day it ceased altogether.

*Case* 7. N. Ferrario presented symptoms quite similar to those of Case 5. The ergot was administered for two days without any effect. Another shop was resorted to, and the ergot now produced the desired operation in 24 hours. It was evident that the medicine was bad in the first instance.

*Case* 8. A female experienced a fall in the sixth month of pregnancy, and aborted. In a month after this a profuse menorrhagia occurred, with the usual pains, &c. The ergot was prescribed, and in four days there was an end of the complaint.

In another short article we shall present to our readers the efficacy of the ergot in hæmorrhages from various parts of the body, as promised in a subsequent communication from the same authors.

